# Lower limb re-vascularization based on the angiosome concept with immediate-early local flap reconstruction: a case report

**DOI:** 10.1093/jscr/rjac320

**Published:** 2022-08-11

**Authors:** Yu Ming Lai, Kae-Sian Tay, Shaun Qing Wei Lee, Allen Wei-Jiat Wong

**Affiliations:** Department of Plastic, Reconstructive and Aesthetic Surgery, Singapore General Hospital, Singapore, Singapore; Department of Orthopaedic Surgery, Singapore General Hospital, Singapore, Singapore; Department of Vascular Surgery, Singapore General Hospital, Singapore, Singapore; Department of Plastic, Reconstructive and Aesthetic Surgery, Seng Kang General Hospital, Singapore, Singapore

## Abstract

Lower extremity wounds are a healthcare issue that can result in debilitating consequences. Peripheral arterial occlusive disease (PAOD) being a major contributing factor to the disease, advance revascularization procedures (Angioplasty) based on the angiosome concept has been established in the literature to improve blood supply and promote better healing outcomes. We present a case of a 59-year-old lady with background of hypertension, diabetes and PAOD, was diagnosed with a non-healing foot wound with exposed metal implant. She had targeted angioplasty done based on angiosome concept followed by early local flap reconstruction for coverage, which healed well without complications. Although free tissue transfer has now been the mainstay for lower limb reconstruction with the advancement in microsurgery. Local flaps remain in the armamentarium of lower extremity reconstruction with small to medium sized wound defects. This case demonstrates the advantage of proceeding with immediate-early local flap reconstruction following successful targeted re-vascularization.

## INTRODUCTION

Lower extremity wound is a major issue in healthcare especially in the elderly population that can hugely impact not only the patient’s health but socially and economically as well. An issue commonly due to diabetic foot ulcers, peripheral vascular diseases, trauma, large tumor resection and pressure sores [1, 2]. Wounds of the lower extremity can occur in varying degrees ranging from small, simple wounds to large, complex wound defects with exposed critical structures or metal implants [1, 2]. This can potentially lead to debilitating complications and eventual limb amputation. With that, lower limb wound management and reconstruction remains a challenge for reconstructive surgeons [1, 3].

However, with better anatomical understanding, improved reconstructive techniques and advanced re-vascularization procedures (Bypass or Angioplasty) with a multidisciplinary management approach, outcomes of lower limb wound healing and successful limb salvage has improved [1, 2]. We present a case of a patient who underwent re-vascularization with subsequent soft tissue reconstruction for a foot wound.

## CASE PRESENTATION

**Figure 1:**
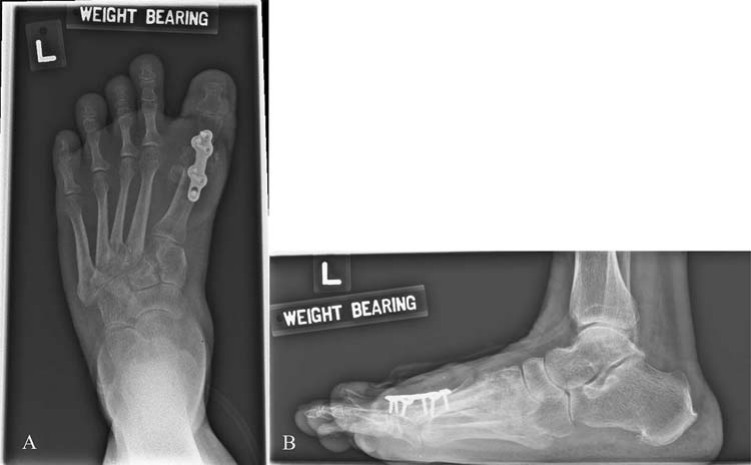
AP (**A**) and Lateral (**B**) X-ray the left foot. Metal implants seen over dorsal MTPJ of the 1st toe after corrective hallux valgus surgery.

**Figure 2:**
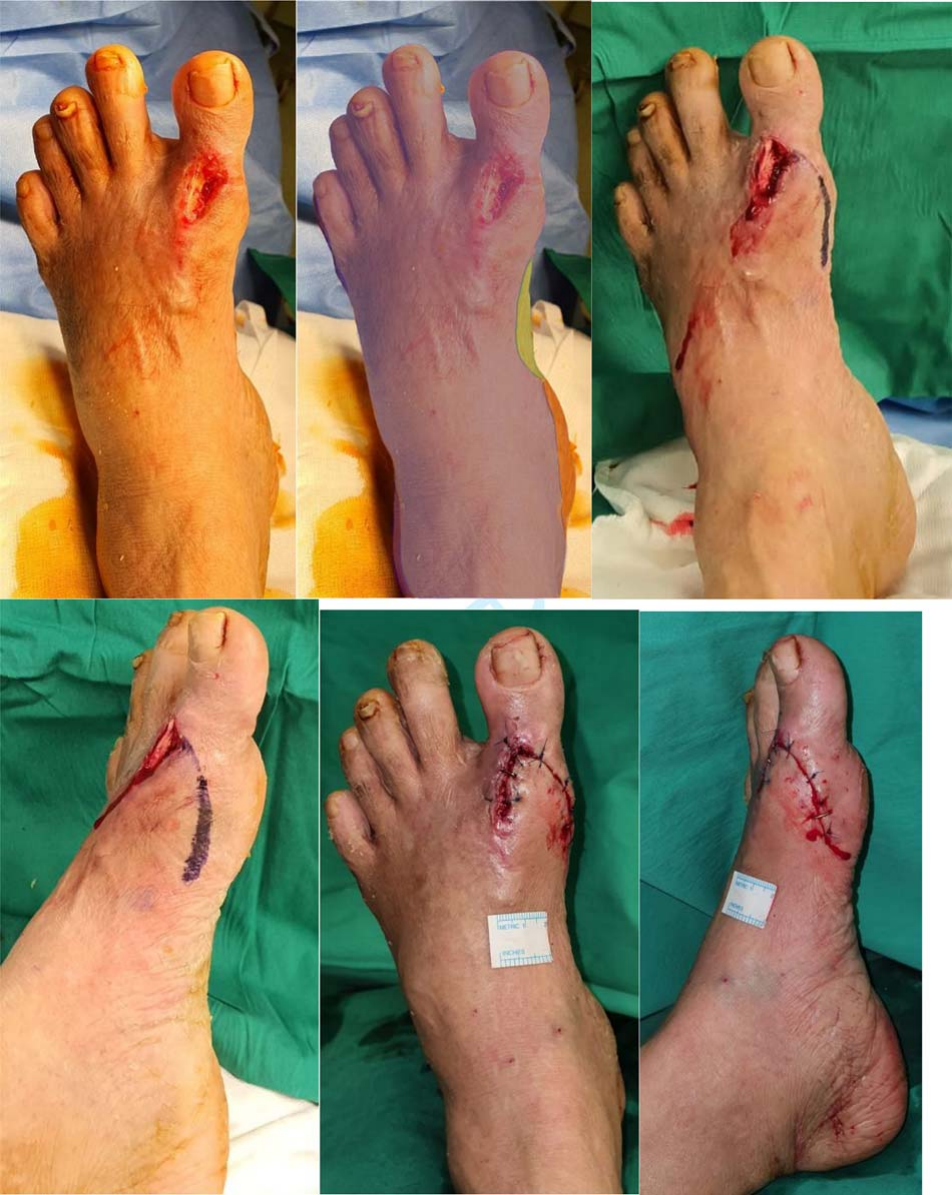
(**A**) 4 × 2cm full thickness wound defect over dorsal MTPJ of left 1st toe with underlying exposed tendon (EHL) and metal implant. (**B**) Angiosome of the foot—ATA [purple]; medial plantar artery [green]; calcaneal branch of PTA (orange) (**C**, **D**) Images showing the incision markings for a medially-based design rotation flap. (**E**, **F**) Images showing the final inset of the medially-based rotation flap to the defect site.

**Figure 3:**
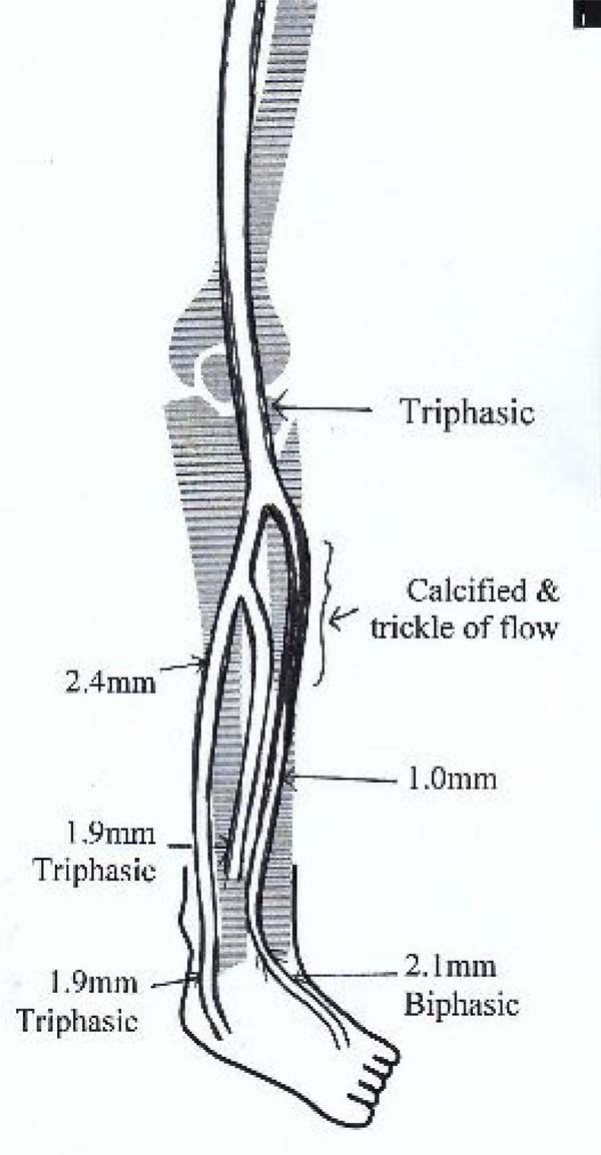
Illustrated diagram of left lower limb arterial duplex scan showing 70–80% occlusive disease at the proximal ATA.

Mdm. S.N.C is a 59-year-old lady with a background of hypertension and diabetes mellitus who had corrective surgery for her left hallux valgus deformity in July 2020 (Fig. 1). This was complicated by wound dehiscence and exposure of the underlying tendon (Extensor hallucis longus) and implant (Fig. 2). An arterial duplex scan was done, which showed 70–80% occlusive disease over the proximal anterior tibial artery (ATA; Fig. 3) that likely contributed to her poor wound healing.

**Figure 4:**
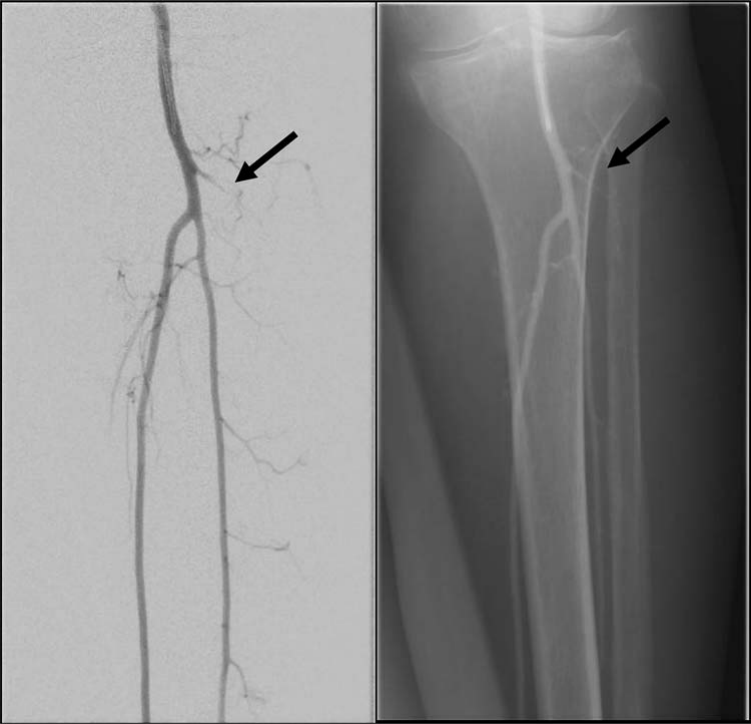
Left lower limb diagnostic angiogram showing occlusion of the ATA with poor flow (arrow).

**Figure 5:**
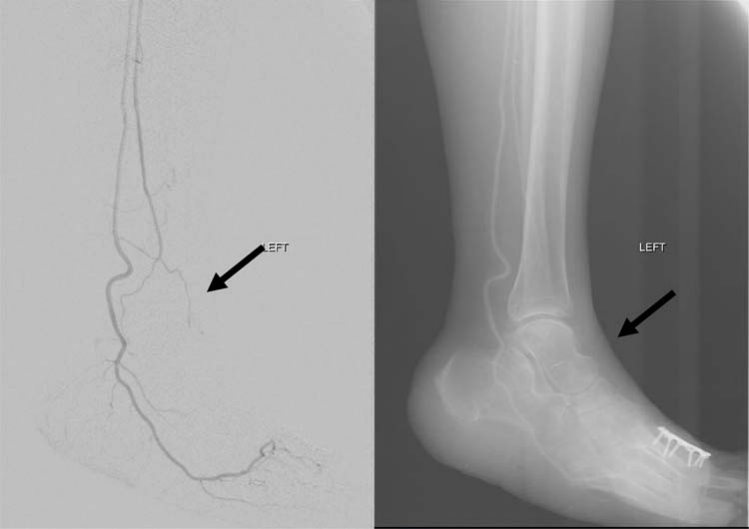
Angiogram of left distal leg and foot showing poor flow of the distal ATA and dorsalis pedis artery (arrow).

**Figure 6:**
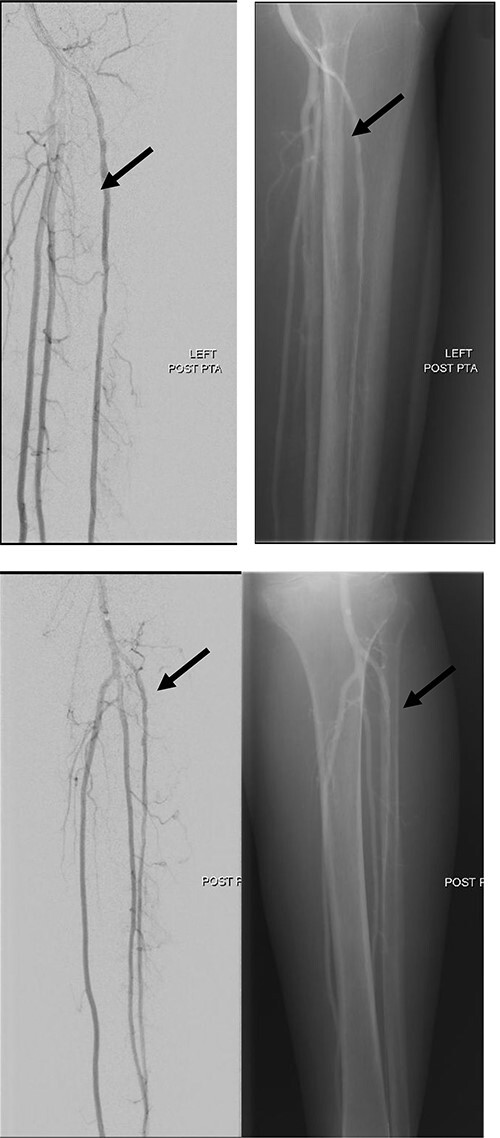
Good arterial flow of the ATA (arrow) after successful angioplasty and stenting.

**Figure 7:**
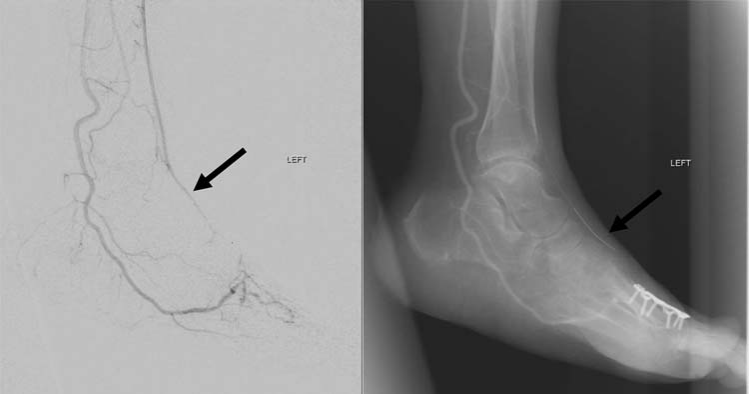
Arterial flow noted at distal ATA and dorsalis pedis artery (arrow) after successful angioplasty and stenting.

She subsequently underwent left lower limb angioplasty and stenting of the ATA with good results (Figs 4–7) and was started on antiplatelet therapy. Following successful angioplasty, she underwent early wound coverage procedure. A medially based rotation advancement flap was raised for coverage of wound defect. Patient completed 2 weeks course of broad-spectrum antibiotics post-operatively. Patient’s flap was stable and she was allowed partial weight bear over her left lower limb with Darco shoes at post-operative Day 12.

**Figure 8:**
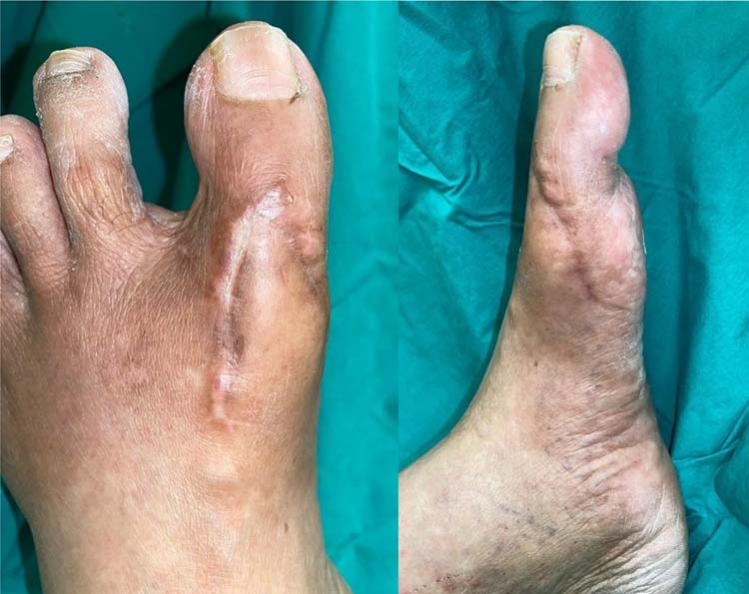
Three-month post-operative, flap is fully healed and left foot remains well vascularized with palpable dorsalis pedis pulse.

At 4 weeks post-operative date, her wound/flap has fully healed. She was then allowed full weight bearing over her left lower limb. At 3 months post-operative date, flap was fully healed, and her left lower limb remained well vascularized (Fig. 8).

## DISCUSSION

Wound healing comprises of a complex regulated pathway that occurs in three phases: hemostasis, inflammatory phase, proliferative phase and maturation/remodeling phase.

There are various factors that can disrupt this process and lead to wound breakdown or dehiscence. Factors include local factors such as wound site infection, high tension skin closure, edema/hematoma and ischemia; and systemic factors, which includes smoking, malnutrition, chronic diseases—example diabetes mellitus, chronic kidney diseases, obesity, connective tissue diseases and medications such as steroids and immunosuppressants [4–6].

Zolper *et al.* reviewed patients who had lower limb orthopedic surgery with wound dehiscence, 47% had associated arterial abnormality on angiography and ~53% of those individuals had arterial abnormality based on the angiosome of the wound defect location [4]. Notably in this case, poor perfusion as a result from stenosis of the proximal ATA, likely contributed to her surgical wound complication. With that, knowledge of lower limb vasculature and understanding its dynamic nature is crucial. The angiosome concept, which is defined as a composite unit of tissue that is fed by a named or source artery, was described by Ian Taylor, plays an important role in managing lower limb wounds associated with peripheral vascular disease [7–9].

Attinger further elaborated on angiosomes below the knee region. He described the six angiosomes of the foot and ankle region that is supplied by three main source arteries; the posterior tibial artery (PTA) that supplies the medial ankle and plantar foot, the peroneal artery to the anterolateral ankle and lateral heel and the ATA to the dorsum of foot [10, 11]. Our patient’s arterial abnormality at the ATA, correlated with her wound location based on the lower limb angiosomes.

Evidence in current literature suggest that revascularization based on the angiosome concept, improves overall wound outcomes and increase limb salvage rates. Alexandrescu *et al*. found that the angiosome-targeted revascularization achieved better healing outcomes and higher limb salvage rates than non-angiosome targeted revascularization [12]. In addition, Zheng *et al*. noted that direct angiosomal angioplasties has lower rates of unhealed ulcer as compared to indirect revascularization (31.7% vs 83.4%) and with higher limb salvage rate (89.2% vs 70.4%) [13].

Targeted revascularization was performed in this case based on the angiosome that correlated with the location of her wound increase blood delivery, improving healing potential and ability to combat infection. With the exposed metal implant, flap (Local or Free) coverage will be necessary and with consideration of the defect size and location, a local rotational flap reconstruction was performed as described above.

Combined re-vascularization with subsequent reconstruction of the lower limb with free flaps has been described in numerous articles [14]. A retrospective study by Chou on patients who underwent re-vascularization procedure showed an overall flap success rate of 90% with good limb salvage outcomes [15]. Although majority of the studies focuses on free tissue transfer, which is the mainstay for soft tissue reconstruction in the distal third of the leg and foot, local flaps remain part of the armamentarium for lower limb reconstruction especially for small wound defects as they can be technically less demanding, lower donor site morbidity and single-staged with short operative time while achieving good functional and aesthetic outcome as described by AlMugaren [3].

In conclusion, this case report displays the potential advantage of approaching small wound defects of the foot and ankle region with peripheral arterial disease, as a combination procedure with targeted re-vascularization based on the angiosome concept followed by immediate local flap reconstruction.

## CONFLICT OF INTEREST STATEMENT

The authors have no conflicts of interest to declare. All co-authors have seen and agree with the contents of the manuscript and there is no financial interest to report.

## FUNDING

None.
